# Predictive role of endothelin in left ventricular remodeling of chronic kidney disease

**DOI:** 10.1080/0886022X.2018.1455586

**Published:** 2018-03-28

**Authors:** Tao Peng, Xia Li, Zhao Hu, Xiangdong Yang, Chengjun Ma

**Affiliations:** a Department of Nephrology, Shandong University Qilu Hospital, Jinan, P. R. China;; b Xiying Town Hospital, Licheng District, Jinan, P. R. China;; c Department of Nephrology, Shandong University Qilu Hospital (Qingdao), Qingdao City, P. R. China

**Keywords:** Chronic kidney disease, ventricular remodeling, endothelin-1, cardiac complication, non-dialysis

## Abstract

**Background:** In chronic kidney disease (CKD), endothelin-1 (ET-1) always increases and there are changes in cardiac ultrasonography. In the present study, we aimed at investigating the role of serum ET-1 in predicting cardiac complications in patients with CKD.

**Methods:** The level of serum ET-1 was measured by enzyme-linked immunosorbent assay (ELISA) and cardiac ultrasonography was performed in enrolled patients. Indexes of heart failure, such as left ventricular mass index, interventricular septum thickness and left ventricular end diastolic diameter, were measured in patients with CKD.

**Results:** In the present study, we found that the level of serum ET-1 was significantly correlated with left ventricular mass index, interventricular septum thickness and left ventricular end diastolic diameter (*p* < .001) in non-dialysis patients with CKD.

**Conclusions:** The results of the present study indicated that the level of serum ET-1 is closely related to the cardiac complications of CKD and is a useful predictor of cardiac complication.

## Introduction

Chronic kidney disease (CKD) is one of the major causes of death in patients with kidney disease. Early detection and active treatment of its complications, especially cardiovascular complications, can significantly improve the prognosis of patients. In the present study, we observed changes in serum endothelin-1 (ET-1) levels and ultrasonographic parameters reflecting structural and functional changes in non-dialysis patients with CKD. The aim of the present study was to investigate the role of serum ET-1 in the prediction of cardiac complications in patients with CKD.

## Materials and methods

### Materials

One hundred seventeen patients with CKD without dialysis were enrolled in the present study at the Department of Nephrology, Qilu Hospital of Shandong University from July 2014 to April 2016. All met the diagnostic criteria for CKD. All enrolled patients were divided into five stages according to the US NKF-K/DOQI working group recommendations in 2003 [1]: (see Table 1)

**Table 1.** Level of ET-1 in enrolled patient with CKD ($\bar{x}$ ± s).

Stage	*n*	M/F	Average age (y)	GFR (ml/min·1.73 m^2^)	ET-1 (*ρ*/ng·L^−1^)
I	19	11/8	43.26 ± 11.56	≥90	59.94 ± 14.88
II	25	11/14	46.48 ± 12.23	60–89	102.49 ± 18.01*
III	23	12/11	48.95 ± 14.91	30–59	154.51 ± 47.69*&
IV	27	16/11	47.56 ± 12.58	15–29	189.15 ± 43.16*&Δ
V	23	11/12	49.68 ± 12.63	<15	190.39 ± 42.51*&Δ

M: male; F: female; ET-1: Endothelin-1; GFR: glomerular filtration rate.

* *p <* .01 vs. Stage I.

& *p <* .01 vs. Stage II.

Δ *p <* .01 vs. Stage III.

Stage I, glomerular filtration rate (GFR) ≥ 90 mL/(min·1.73 m^2^), 19 cases, 11 males and 8 females, average age (43.26 ± 11.56) years;
Stage II, GFR 60–89 mL/(min·1.73 m^2^), 25 cases, 11 males and 14 females, average age (46.48 ± 12.23) years;
Stage III, GFR 30–59 mL/(min·1.73 m^2^), 23 cases, 12 males and 11 females, average age (48.95 ± 14.91) years;
Stage IV, GFR 15–29 mL/(min·1.73 m^2^), 27 cases, 16 males and 11 females, average age (47.56 ± 12.58) years;
Stage V, GFR <15 mL/(min·1.73 m^2^), 23 cases, 11 males and 12 females, average age (49.68 ± 12.63) years.

### Equipment

The equipment used was as follows:

γ Radioimmunoassay (Technology Industrial Corporation, University of Science and Technology of China), HP Sonos 5500 color Doppler echocardiography (Philips Corporation, Netherlands), LD24-0.8 Automatic balance microcentrifuge (Beijing Medical Centrifuge Factory), 772 grating spectrophotometer (Shanghai No. 3 Analytical Instrument Factory), and Bayer l650 automatic biochemical analyzer (Bayer Corporation, Germany). The ET-1 kit was provided by Nanjing Jiancheng Bioengineering Research Institute, with intra-assay variation CV <10% and inter-assay variation CV <15%.

## Methods

### Endothelin-1

Six milliliters of venous blood was collected from the left elbow median artery from all patients with a fasting status in the morning (at least 8 h without eating). Serum was separated from 2 mL of venous blood and was stored by cryopreservation, and the level of ET-1 in the serum was measured from these samples. The remaining 4 mL of venous blood was evenly injected into glass anticoagulant test tubes, 2 mL each. Serum urea nitrogen, creatinine and hemoglobin were measured by enzymatic methods the picric acid method and the high iron oxide method. Medication with an angiotensin-converting enzyme inhibitor (ACEI), an Angiotensin II receptor antagonist (ARB), calcium channel blockers (CCB), diuretics, nitrates, digitalis drugs and vasodilators was excluded from all enrolled patients at least 36–48 h before blood samples were collected.

### Cardiac ultrasonography

Left ventricular end-diastolic diameter (LVDd), interventricular septal thickness (IVST) and left ventricular posterior wall thickness (LVPWT) were measured from the standard sternal left ventricular long axis, according to the American Society of Echocardiography (American Society of Echocardiography) standard by HP Sonos 5500 color Doppler echocardiography. The left ventricular ejection fraction (EF) was calculated according to Simpson7S. The left ventricular mass index (LVMI) and relative wall thickness (RWT) were calculated according to Devereux’s calibration formula. All enrolled patients were measured for height (H, cm), weight (W, kg), systolic blood pressure (SBP), diastolic blood pressure (DBP), hemoglobin (Hgb), and age on admission to the present study. We used the following formula (body surface area (BSA) = 0.0061 × *H* + 0.0128 × *W* − 0.1529) to calculate the body surface of enrolled patients. Glomerular filtration rate (GFR) was calculated using the modification of diet in renal disease (MDRD) [2–6].

$$\text{LVM}\left(\text{g}\right)=0.\text{8}\times \left\{\text{1}.0\text{4}\times \left[{\left(\text{LVDd}+\text{IVST}+\text{LVPWT}\right)}^{\text{3}}-{\text{LVDd}}^{\text{3}}\right]\right\}+0.\text{6}$$

BSA = 0.0061×H+0.0128×W-0.1529

$$\text{LVMI }\left(\text{g}/{\text{m}}^{\text{2}}\right)\;=\text{ LVM}/\text{BSA}$$

RWT=2LVPWT/LVDd

### Outcome criteria

We used echocardiography normal references from Ruijin Hospital, Shanghai Second Medical University, Institute of Hypertension. Normal standards of LVDd, IVST, and LVPWT were 40–60, 6–11, and 6–11 mm, respectively. EF < 50% and E/A < 1 are diagnosed as left ventricular systolic dysfunction and left ventricular diastolic dysfunction. For males and females, the diagnostic standard for left ventricular hypertrophy is different: in males it is LMVI ≥135 g/m^2^ and in females it is LVMI ≥111 g/m^2^. RWT > 0.45 was the standard for cardiac enlargement [7,8]. Left ventricular configurations were divided into four types according to RWT and LVMI: Left ventricular hypertrophy classifications: centripetal hypertrophy, centripetal reconstruction, eccentric hypertrophy and normal structure of the four types [9].

### Statistical analysis

Data were expressed as the mean ± SD ($\bar{x}$ ± s). Statistical analyzes were evaluated using SPSS for Windows 16.0 software, followed by a one-way analysis of variance and the *q*-test to assess the significance of the differences among the various groups. Correlation analyzes were assessed by a linear regression analysis. Significance was considered *p* < .05.

## Results

### Left ventricular configuration distribution

Of the 117 patients, left ventricular hypertrophy was found in 59 patients (50.63%), including left ventricular concentric hypertrophy in 22 patients (18.75%) and left ventricular eccentric hypertrophy in 37 patients (31.88%). Twenty-seven patients had left ventricular concentric, accounting for 23.13%, and 31 cases that had a normal structure, accounting for 26.25%.

The levels of ET-1 and the results of cardiac ultrasonography are seen in Tables 1 and 2. The correlation between endothelin and echocardiographic indexes is seen in Table 3 [10]. A multiple stepwise regression analysis was performed on multiple factors affecting left ventricular hypertrophy. The results showed that LVMI was positively correlated with systolic blood pressure (SBP) and serum ET-1 activity, and the coefficient of determination was *R*^2^ = 0.408, *F* = 12.819, and *p* = .000 (Table 4).

**Table 2.** Comparison in echocardiographic indicators ($\bar{x}$ ± s).

	Stage 1	Stage 2	Stage 3	Stage 4	Stage 5
LVDd (d/mm)	42.12 ± 4.23	45.10 ± 5.52Δ	49.43 ± 5.39Δ	49.17 ± 5.13Δ	49.76 ± 4.52Δ
IVST (δ/mm)	11.23 ± 2.14	11.38 ± 2.12	12.91 ± 1.68Δ	11.75 ± 1.19	12.98 ± 2.42Δ
LVPWT (δ/mm)	9.61 ± 0.69	9.20 ± 1.88	11.20 ± 1.49*	11.08 ± 1.64*	11.65 ± 3.46*
LVMI (g/m^2^)	95.24 ± 28.67	129.2 ± 41.64Δ	149.96 ± 40.88Δ*	150.34 ± 41.78Δ*	149.91 ± 36.87Δ*
EF (%)	0.65 ± 0.06	0.59 ± 0.06Δ	0.65 ± 0.08*	0.60 ± 0.14Δ	0.59 ± 0.11Δ
E/A	0.94 ± 031	0.98 ± 0.42	0.92 ± 0.12*	0.65 ± 0.35Δ*	0.59 ± 0.25Δ*

Δ *p <* .01 vs. Stage 1.

* *p <* .01 vs. Stage 2.

**Table 3.** Correlation analysis of ET-1 and left ventricular changes^a^.

*x*	*y*	*r*	*p*
ET-1	LVDd	0.331	.029
ET-1	IVST	0.429	.001
ET-1	LVPWT	0.254	.110
ET-1	LVMI	0.494	.000
ET-1	EF	0.36	.824
ET-1	E/A	−0.013	.935

a Peng et al. [10].

**Table 4.** Multivariate regression analysis.

Regression equation	*R^2^*	*F*	*p*
LVMI =49.31 + 1.003 × SBP +0.327 × ET-1	0.408	12.819	.000

## Discussions

Chronic kidney disease is one of the leading public health issues due to frequent and serious complications. Once the function of the kidneys is disrupted, regardless of the etiology, there are numerous factors that can accelerate the decrease in glomerular filtration rate, including hypertension, proteinuria and dyslipidemia. Patients with CKD are at high risk for cardiovascular diseases [11]. Cardiovascular complications are a common complication of CKD, accounting for approximately 50% of deaths in patients with CKD, and the incidence of left ventricular hypertrophy is high. There is an endothelial dysfunction in CKD, and the manifestation of the endothelial dysfunction is the imbalance of the various factors deriving from endothelial cells, mainly the imbalance of endothelium-derived vasoconstrictor factors (e.g., ET-1) and relaxation factors (e.g., NO) [10,12].

Endothelin-1 is a 21 amino acid peptide produced by a variety of cell types throughout the body. ET-1 was originally identified as a vasoconstrictor with activity at sub-nanomolar concentrations, and it is now additionally known to be a pro-inflammatory, mitogenic, natriuretic, and nociceptive mediator [13]. Under healthy conditions, ET-1 is constitutively expressed in all cells of the glomerulus and participates in homeostasis of the glomerular structure and filtration function. ET-1 is the most potent and long-lasting vasoconstrictor known, being 100-times more potent than noradrenaline. ET-1 was detected as a peptide with vasoconstrictor activity in the supernatant of endothelial cells [14]. The present study showed that with the deterioration of renal function in patients with CKD, serum ET-1 activity gradually increased, and there were significant differences among different stages. The mechanism of elevated ET-1 may be due to residual glomerular vascular blood flow redistribution, which can cause changes in microvascular endothelial cell shear stress, proendothelin gene expression, and increased synthesis and release of mature ET-1. Glomerular local vasoactive substances, such as angiotensin II, thromboxane A, and transforming growth factor β can stimulate an increase of renal ET-1 biosynthesis. Ischemia, hypoxia, high sodium, high glucose, hyperlipidemia, acid metabolites and mechanical injury increase synthesis and reduce clearance of ET-1. Elevated ET-1 further alters renal hemodynamics, promotes mesangial cell proliferation, and accelerated renal interstitial fibrosis, leading to glomerular sclerosis, which forms a vicious circle, resulting in undesirable consequences.

The results of the present study indicated that there were significant correlations between levels of serum ET-1 and LVMI, IVST, and LVDd in patients with CKD, and there were no significant correlations between levels of serum ET-1 and LVPWT, EF, or E/A. A stepwise regression analysis was performed on multiple factors influencing LVMI. After correction for blood pressure, only ET-1 was associated with LVMI, which suggested that ET-1 causes left ventricular hypertrophy independent of blood pressure.

The present study indicated that the effect of ET-1 on changing the structure of the left ventricle may be related to the following factors: ET-1 can stimulate myocardial fibroblast Type I and Type III collagen proliferation and synthesis and promote myocardial collagen network reconstruction, which plays an important role in myocardial fibrosis and remodeling. ET-1 can induce c-fos gene expression in cardiomyocytes, activate the local renin–angiotensin–aldosterone system (RAAS), and promote proliferation and hypertrophy of cardiomyocytes. ET-1 can also promote platelet-derived growth factor (PDGF), TGF release, and indirectly promote myocardial cell proliferation, which leads to cardiac hypertrophy. Another study [15] found that endothelin receptor antagonists (ETR) can prevent myocardial fibrosis and myocardial cell wall thickening of small arterial blood vessels in uremic rats, confirming that ET-1 is closely related to left ventricular remodeling.

The results of the present study showed that with the deterioration of renal function, LVMI increased gradually in the different groups, and there was a significant correlation between LVMI and ET-1 (*p < *.01). It was confirmed that with renal function deterioration in chronic renal failure, the appearance of hypertension, accumulation of uremic toxins, and imbalance in endothelial function became increasingly serious. The patient’s cardiac structure and function deteriorated, and of all the indicators of cardiac ultrasound, in particular, LVMI and IVST were closely related to ET-1 activity. So, perhaps we can partially predict developing complications of CKD, especially cardiovascular complications, thereby reducing mortality and improving survival and quality of life in patients with CKD.

In summary, cardiovascular complications are a common complication of CKD, and with the deterioration of renal function in patients with CKD, serum ET-1 levels gradually increased. The level of serum ET-1 is closely related to cardiac complications of CKD.

## Limitations

This study had some limitations, including the small sample size and the short time.

## References

[1] Rizzetto F, Leal VO, Bastos LS, et al Chronic kidney disease progression: a retrospective analysis of 3-year adherence to a low protein diet. Ren Fail. 2017;39:357–362. 28152653 10.1080/0886022X.2017.1282374 PMC6014383

[2] David-Neto E, Kamada Triboni AH, Ramos F, et al Evaluation of MDRD4, CKD-EPI, BIS-1, and modified Cockcroft–Gault equations to estimate glomerular filtration rate in the elderly renal-transplanted recipients. Clin Transplant. 2016;30:1558–1563. 27726196 10.1111/ctr.12857

[3] Dsimone G, Daniels SR, Devereux RB, et al Left ventricular mass and body size in normotensive children and adulrs: assessment of allometric relations and impact of overweight. J Am Coll Cardiol. 1992;20:1251–1260. 1401629 10.1016/0735-1097(92)90385-z

[4] Yao T. Physiology. 5th ed Beijing: People’s Medical Publishing House; 2000 p. 215–216.

[5] Palmieri V, Bella JN, Arnett DK, et al Effect of type 2 diabetes mellitus on left ventricular geometry and systolic function in hypertensive subjects. Circulation. 2001;103:103–107. 10.1161/01.cir.103.1.102 11136693

[6] Lin J, Wang F, Weiner RB, et al Blood pressure and LV remodeling among American-style football players. JACC Cardiovasc Imaging. 2016;9:1367–1376. 27931524 10.1016/j.jcmg.2016.07.013 PMC5157690

[7] Cho H, Choi HJ, Kang HG, et al Influence of the method of definition on the prevalence of left-ventricular hypertrophy in children with chronic kidney disease: data from the know-Ped CKD study. Kidney Blood Press Res. 2017;42:406–415. 28689198 10.1159/000478867

[8] Bacharova L, Estes HE, Schocken DD, et al The 4th report of the working group on ECG diagnosis of left ventricular hypertrophy. J Electrocardiol. 2017;50:11–15. 27890283 10.1016/j.jelectrocard.2016.11.003

[9] Vetta F, Cicconetti P, Ronzoni S, et al Hyperinsulinaemia, regional adipose tissue distribution and left ventricular mass in normotensive, elderly, obese subjects. Eur Heart J. 1998;19:326–331. 9519328 10.1053/euhj.1997.0731

[10] Peng T, Hu Z, Wu L, et al Correlation between endothelial dysfunction and left ventricular remodeling in patients with chronic kidney disease. Kidney Blood Press Res. 2014;39:420–426. 25412643 10.1159/000368455

[11] Zavidić T, Lodeta B, Lovrinić Đ. Use of statins in patients with chronic kidney disease to prevent cardiovascular disease. Acta Med Croatica. 2016;70:301–307. 29087163

[12] Rebić D, Rašić S, Valjevac A, et al Biomarkers of cardiovascular remodeling in patients on peritoneal dialysis. Am J Nephrol. 2014;39:92–99. 24503489 10.1159/000358261

[13] Fox BM, Kasztan M. Endothelin receptor antagonists in sickle cell disease: a promising new therapeutic approach. Life Sci. 2016;159:15–19. 27049871 10.1016/j.lfs.2016.04.001 PMC4992628

[14] Barton M, Sorokin A. Endothelin and the glomerulus in chronic kidney disease. Semin Nephrol. 2015;35:156–167. 25966347 10.1016/j.semnephrol.2015.02.005 PMC4731878

[15] Woodell TB, Hughes-Austin JM, Tran TV, et al Associations between cystatin C-based eGFR, ambulatory blood pressure parameters, and in-clinic versus ambulatory blood pressure agreement in older community-living adults. Blood Press Monit. 2016;21:87–94. 26683379 10.1097/MBP.0000000000000168 PMC4783213

